# Cotargeting histone deacetylases and oncogenic BRAF synergistically kills human melanoma cells by necrosis independently of RIPK1 and RIPK3

**DOI:** 10.1038/cddis.2013.192

**Published:** 2013-06-06

**Authors:** F Lai, S T Guo, L Jin, C C Jiang, C Y Wang, A Croft, M N Chi, H-Y Tseng, M Farrelly, B Atmadibrata, J Norman, T Liu, P Hersey, X D Zhang

**Affiliations:** 1 School of Medicine and Public Health, University of Newcastle, Callaghan, NSW, Australia; 2 Department of Molecular Biology, Shanxi Cancer Hospital and Institute, Taiyuan, Shanxi, People's Republic of China; 3 Oncology and Immunology Unit, Calvary Mater Newcastle Hospital, Waratah, NSW, Australia; 4 Children's Cancer Institute Australia for Medical Research, The University of New South Wales, Sydney, NSW 2052, Australia; 5 Electron Microscope Unit, Mark Wainwright Analytical Centre, The University of New South Wales, Sydney, NSW 2052, Australia; 6 Kolling Institute for Medical Research, University of Sydney, St. Leonards, NSW, Australia

**Keywords:** histone deacetylase inhibitors, BRAF inhibitors, melanoma, necrosis

## Abstract

Past studies have shown that histone deacetylase (HDAC) and mutant BRAF (v-Raf murine sarcoma viral oncogene homolog B1) inhibitors synergistically kill melanoma cells with activating mutations in BRAF. However, the mechanism(s) involved remains less understood. Here, we report that combinations of HDAC and BRAF inhibitors kill BRAF^V600E^ melanoma cells by induction of necrosis. Cotreatment with the HDAC inhibitor suberoylanilide hydroxamic acid (SAHA) or panobinostat (LBH589) and the BRAF inhibitor PLX4720 activated the caspase cascade, but caspases appeared dispensable for killing, in that inhibition of caspases did not invariably block induction of cell death. The majority of dying cells acquired propidium iodide positivity instantly when they became positive for Annexin V, suggesting induction of necrosis. This was supported by caspase-independent release of high-mobility group protein B1, and further consolidated by rupture of the plasma membrane and loss of nuclear and cytoplasmic contents, as manifested by transmission electron microscopic analysis. Of note, neither the necrosis inhibitor necrostatin-1 nor the small interference RNA (siRNA) knockdown of receptor-interacting protein kinase 3 (RIPK3) inhibited cell death, suggesting that RIPK1 and RIPK3 do not contribute to induction of necrosis by combinations of HDAC and BRAF inhibitors in BRAF^V600E^ melanoma cells. Significantly, SAHA and the clinically available BRAF inhibitor vemurafenib cooperatively inhibited BRAF^V600E^ melanoma xenograft growth in a mouse model even when caspase-3 was inhibited. Taken together, these results indicate that cotreatment with HDAC and BRAF inhibitors can bypass canonical cell death pathways to kill melanoma cells, which may be of therapeutic advantage in the treatment of melanoma.

Although mutant BRAF (v-Raf murine sarcoma viral oncogene homolog B1) inhibitors such as vemurafenib and dabrafenib have achieved unprecedented clinical responses in the treatment of melanomas with activating mutations in BRAF, complete remission is rare and a proportion of mutant BRAF melanomas are less responsive to the inhibitors.^1, 2, 3, 4^ On the other hand, durations of responses are commonly limited with most patients relapsing within 1 year, indicative of development of acquired drug resistance.^1, 2, 3, 4^ Moreover, it has been recently shown that vemurafenib-resistant mutant BRAF melanoma cells may become drug-dependent for their continuous proliferation.^5^

Multiple mechanisms have been shown to contribute to BRAF inhibitor resistance in melanoma cells.^1, 2, 3, 4^ These include those leading to insufficient inhibition of MEK/extracellular signal-regulated kinase (ERK) signaling and those promoting melanoma cell survival and proliferation alternative to the MEK/ERK pathway, such as increased activation of the PI3K/Akt or NF-*κ*B pathway.^6, 7, 8, 9, 10, 11^ Indeed, combinations of BRAF inhibitors and inhibitors of MEK, such as trametinib, necessary to further inhibit MEK/ERK signaling have yielded promising results in clinical trials.^12, 13, 14^ Co-targeting the PI3K/Akt and MEK/ERK pathways is also being evaluated in early clinical studies.^9, 15^ In addition, inhibition of HSP90, a chaperon involved in regulating conformation of many kinases including mutant BRAF and Akt, has been demonstrated to overcome BRAF inhibitor resistance in melanoma cells.^16^

Our past results have suggested that sensitivity to induction of cell death may be a major determinant of long-term responses of BRAF^V600E^ melanoma cells to BRAF inhibitors.^10^ Killing of melanoma cells by BRAF or MEK inhibitors involves regulation of anti- and prosurvival proteins of the Bcl-2 family, in particular, Bim and Mcl-1.^17, 18, 19, 20^ However, induction of melanoma cell death by inhibition of MEK has been shown to be caspase-independent, although the caspase cascade is activated upon MEK inhibition in sensitive cells.^21^

Histone deacetylase (HDAC) inhibitors are emerging as a promising class of compounds in the treatment of cancer with low *in vivo* side-effect profiles.^22, 23^ Although monotherapy with HDAC inhibitors is not superior to dacarbazine (DTIC) in the treatment of melanoma,^24, 25^ combinations of HDAC inhibitors and other therapeutic agents are currently being evaluated.^26, 27^ Similar to cell death induced by inhibition of BRAF or MEK, induction of melanoma cell death by HDAC inhibitors involves regulation of various Bcl-2 family proteins including Bim and Mcl-1.^28, 29^ Furthermore, HDAC inhibitors such as suberoylanilide hydroxamic acid (SAHA) can also induce caspase-independent cell death^30, 31^

While induction of apoptosis is an important mechanism responsible for killing of cancer cells by many therapeutic drugs, increasing evidence indicates that programmed necrosis also contributes to cell death induced by various stimuli such as genotoxic stress and activation of death receptors.^32, 33^ Although signaling pathways leading to programmed necrosis have not been well-defined, it is known that activation of receptor-interacting protein kinase 1 (RIPK1) and RIPK3 is required for the transduction of necrotic signaling in many experimental systems.^32, 33^ Once activated, RIPK3 recruits and phosphorylates mixed lineage kinase domain-like (MLKL), leading to necrosis reportedly by sequential activation of the mitochondrial protein phosphatase PGAM5 and the mitochondrial fission factor Drp1.^34, 35^

We have previously shown that the HDAC inhibitor SAHA and the BRAF inhibitor PLX4720 synergistically induce cell death in BRAF^V600E^ melanoma cells.^36^ In this study, we have examined more closely the mode of BRAF^V600E^ melanoma cell death induced by combinations of HDAC and BRAF inhibitors. We report here that although cotreatment with HDAC and BRAF inhibitors activates the caspase cascade and the mitochondrial apoptotic signaling, it kills BRAF^V600E^ melanoma cells predominantly by induction of necrosis in a RIPK1- and RIPK3-independent manner. In addition, we demonstrate that SAHA and the clinically available BRAF inhibitor vemurafenib cooperatively inhibit BRAF^V600E^ melanoma xenograft growth in a mouse model.

## Results

### Synergistic induction of BRAF^V600E^ melanoma cell death by HDAC and BRAF inhibitors is associated with activation of the caspase cascade and damage to the mitochondria

**Figure 1:**
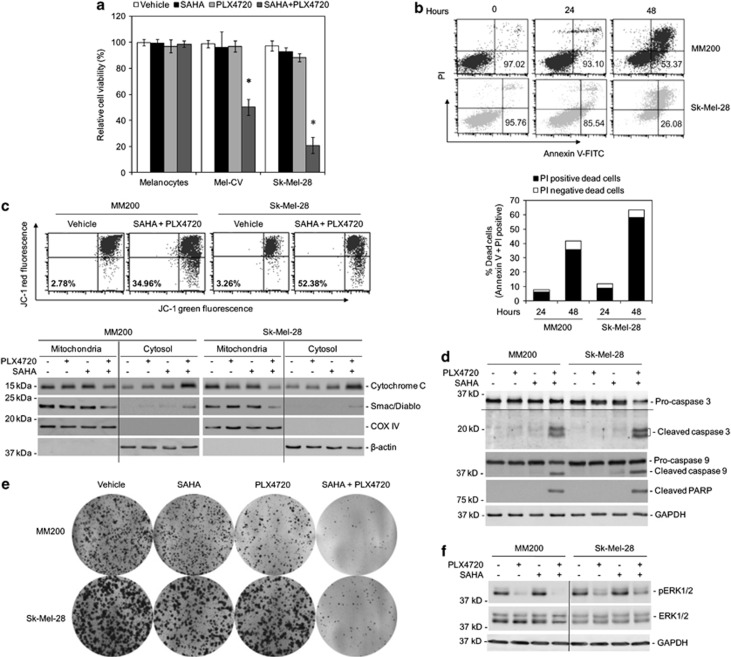
Killing of BRAF^V600E^ melanoma cells by cotreatment with SAHA and PLX4720 is associated with activation of the caspase cascade and damage to the mitochondria. (**a**) HEMn-MP melanocytes, Sk-Mel-28, and Mel-CV melanoma cells treated with the vehicle control (DMSO), SAHA (2 *μ*M), PLX4720 (5 *μ*M), or the combination of SAHA and PLX4720 for 48 h were subjected to CellTiter-Glo assays. The data shown are mean±S.E.M. of three individual experiments. *P<0.01, two-tailed Student's *t*-test. (**b**) Upper panel: MM200 and Sk-Mel-28 cells were cotreated with SAHA (2 *μ*M) and PLX4720 (5 *μ*M) for indicated periods. Induction of cell death was quantitated by the Annexin V-fluorescein isothiocyante (FITC)/propidium iodide (PI) method. The number in each right bottom quadrant represents the percentage of viable cells in each sample. Lower panel: Comparison of the proportion of dead cells with PI uptake and the proportion of dead cells negative for PI as shown in the upper panel. The data shown are representative of three individual experiments. (**c**) Upper panel: MM200 and Sk-Mel-28 cells treated with the vehicle control (DMSO) or the combination of SAHA (2 *μ*M) and PLX4720 (5 *μ*M) for 36 h were subjected to measurement of reduction in the mitochondrial potential using JC-1 staining. The number in each bottom-left quadrant represents the percentage of cells with reduction in the mitochondrial potential. Lower panel: MM200 and Sk-Mel-28 cells were treated with SAHA (2 *μ*M), PLX4720 (5 *μ*M), or the combination of both for 36 h. Cytosolic and mitochondrial fractions were subjected to western blot analysis of cytochrome *C* and Smac/DIABLO. Analysis of *β*-actin and COX IV were included for relative purity of cytosolic and mitochondrial fractions, respectively. The data shown are representative of three individual experiments. (**d**) MM200 and Sk-Mel-28 cells were treated with SAHA (2 *μ*M), PLX4720 (5 *μ*M), or the combination of both for 48 h. Whole-cell lysates were subjected to western blot analysis of caspase-3, caspase-9, the 89 kDa fragment of cleaved PARP (using an antibody that specifically recognizes this fragment), and glyceraldehyde 3-phosphate dehydrogenase (GAPDH) (as a loading control). The data shown are representative of three individual experiments. (**e**) MM200 and Sk-Mel-28 cells were seeded at 1000 cells per well onto 6-well plates as single-cell suspension. After 24 h, SAHA (2 *μ*M), PLX4720 (5 *μ*M), or the combination of both was added into the culture medium. Cells were allowed to grow for 12 days before being fixed with methanol and stained with crystal violet. The data shown are representative of three individual experiments. (**f**) Whole-cell lysates from MM200 and Sk-Mel-28 cells treated with SAHA (2 *μ*M), PLX4720 (5 *μ*M), or the combination of both for 3 h were subjected to western blot analysis of phosphorylated ERK1/2 (pERK1/2), ERK1/2, and GAPDH (as a loading control). The data shown are representative of three individual experiments

Consistent with our previous reports that the HDAC inhibitor SAHA and the BRAF inhibitor PLX4720 synergistically kill BRAF^V600E^ melanoma cells (MM200, IgR3, and Mel-RMu cells),^36^ cotreatment with SAHA and PLX4720 cooperatively killed Mel-CV and Sk-Mel-28 cells that also harbored BRAF^V600E^, as measured using CellTiter-Glo assays (Figure 1a).^34, 35^ In contrast, the combination did not impinge on survival of cultured human melanocytes (HEMn-MP cells) (Figure 1a). Strikingly, when cooperative induction of cell death was confirmed by measurement of Annexin V positivity and PI uptake using flow cytometry in MM200 and Sk-Mel-28 cells, which were not sensitive to killing by either SAHA or PLX4720 alone (Figure 1a),^36^ it was found that the majority of dying (dead) cells became positive for both Annexin V and PI, and some only for PI, even at 24 h when only a small proportion of cells had committed to death (Figure 1b), suggestive of occurrence of necrosis. Nevertheless, cell death was associated with reduction in mitochondrial membrane potential, mitochondrial release of cytochrome *C* and Smac/DIABLO, activation of caspase-9 and -3, and appearance of a 89 kDa band of poly(ADP ribose) polymerase (PARP) in western blotting analysis that was detected with an antibody that specifically recognizes this cleaved PARP fragment,^37^ suggesting induction of apoptosis (Figures 1c and d). Regardless, the combinatorial effect of SAHA and PLX4720 was echoed by enhanced inhibition of long-term survival of MM200 and Sk-Mel-28 cells as shown in clonogenic assays (Figure 1e). Notably, SAHA alone did not impact on the activation of ERK, nor did it affect the inhibition of ERK by PLX4720 (Figure 1f).

Intriguingly, when we detected PARP with an antibody that recognizes its native form and multiple cleaved fragments,^38^ it was found that a ∼50 kDa band conceivably corresponding to a fragment generated by necrotic cleavage of PARP was readily detectable at remarkably higher levels than native PARP in melanoma cells before treatment (Supplementary Figure 1).^38, 39^ Cotreatment with SAHA and PLX4720 increased its levels (Supplementary Figure 1), supporting induction of necrosis by the combination of the inhibitors. However, the cause of this fragment in untreated melanoma cells remains unclear. Its expression at high levels argues against its origin from spontaneous necrosis of melanoma cells. It is likely that PARP is constitutively cleaved in melanoma cells by proteases such as cathepsins without concurrent occurrence of cell death.^38, 39^ Noticeably, a ∼75 kDa band was also detected in melanoma cells, which was similarly increased by cotreatment with SAHA and PLX4720 (Supplementary Figure 1).

The combinatorial effect of inhibition of HDACs and PLX4720 on melanoma cell survival was confirmed by using the HDAC inhibitor panobinostat (LBH589). Similar to SAHA, LBH589 displayed strong synergy with PLX4720 in killing of BRAF^V600E^ melanoma cells (Supplementary Figures 2 and 3),^36^ which was also associated with the activation of caspase-3 and early uptake of PI when cells committed to death (Supplementary Figures 2 and 3).

### Bim is dispensable for synergistic killing of BRAF^V600E^ melanoma cells by SAHA and PLX4720

**Figure 2:**
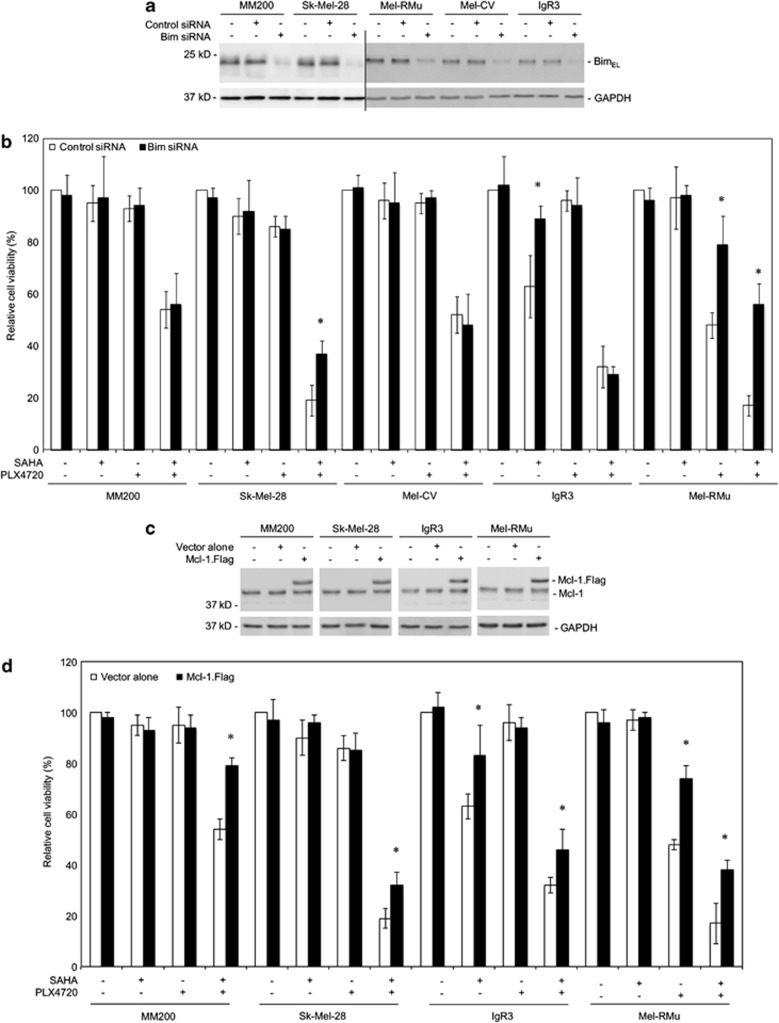
Bim is dispensable for induction of cell death by combinations of SAHA and PLX4720. (**a**) MM200, Sk-Mel-28, Mel-RMu, IgR3, and Mel-CV cells were transfected with the control siRNA and Bim siRNA, respectively. After 24 h, whole-cell lysates were subjected to western blot analysis of Bim and glyceraldehyde 3-phosphate dehydrogenase (GAPDH) (as a loading control). The data shown are representative of three individual experiments. (**b**) MM200, Sk-Mel-28, Mel-RMu, IgR3, and Mel-CV cells were transfected with the control siRNA and Bim siRNA, respectively. After 24 h, cells were treated with SAHA (2 μM), PLX4720 (5 *μ*M), or the combination of both for a further 48 h. Cell viability was measured by CellTiter-Glo assays. The data shown are mean±S.E.M. of three individual experiments. *P<0.01, two-tailed Student's *t*-test. (**c**) MM200, Sk-Mel-28, IgR3, and Mel-RMu cells stably transfected with vector alone or cDNA encoding Flag-tagged Mcl-1 were subjected to western blot analysis of Mcl-1 and GAPDH (as a loading control). The data shown are representative of three individual experiments. (**d**) MM200, Sk-Mel-28, IgR3, and Mel-RMu cells stably transfected with vector alone or cDNA encoding Flag-tagged Mcl-1 were treated with SAHA (2 *μ*M), PLX4720 (5 *μ*M), or the combination of both for 48 h. Cell viability was measured by CellTiter-Glo assays. The data shown are mean±S.E.M. of three individual experiments. *P<0.05, two-tailed Student's *t*-test

Induction of melanoma cell death by HDAC inhibitors or blockade of the RAF/MEK/ERK pathway is associated with the up-regulation of Bim and the downregulation of Mcl-1.^10, 19, 21^ We have also shown previously that the combination of SAHA and PLX4720 further upregulates Bim_EL_.^36^ However, although siRNA knockdown of Bim significantly inhibited reduction in viability of Sk-Mel-28 and Mel-RMu cells induced by cotreatment with SAHA and PLX4720 (*P*<0.05, two-tailed Student's *t*-test), similar to its effect on cell death induced by PLX4720 alone in Mel-RMu cells, and by SAHA alone in IgR3 cells,^17^ it had only a negligible effect on killing of MM200, IgR3, and Mel-CV cells by SAHA plus PLX4720 (<20% inhibition of killing) (Figures 2a and b). These results indicate that Bim is, at least in some BRAF^V600E^ melanoma cells, dispensable for induction of cell death by the combination of SAHA and PLX4720.

We also tested the role of Mcl-1 in regulating sensitivity of BRAF^V600E^ melanoma cells to the combination of SAHA and PLX4720. Overexpression of Mcl-1 inhibited, albeit partially, reduction in cell viability in MM200, Sk-Mel-28, Mel-RMu, and IgR3 cells (Figures 2c and d), suggesting that downregulation of Mcl-1 contributes to synergistic killing of BRAF^V600E^ melanoma cells by the inhibitors irrespective of whether Bim is involved. As anticipated, overexpression of Mcl-1 inhibited reduction in cell viability induced by PLX4720 in Mel-RMu, and by SAHA in IgR3 cells (Figures 2c and d).

### The caspase cascade is dispensable for synergistic killing of BRAF^V600E^ melanoma cells by SAHA and PLX4720

**Figure 3:**
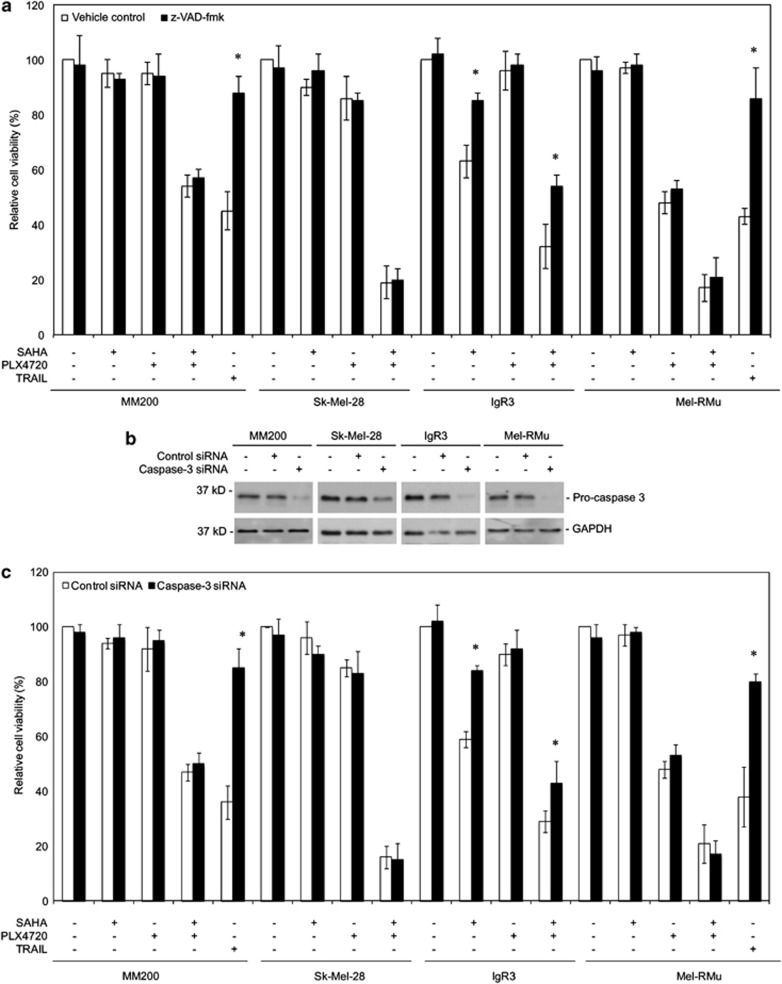
Induction of cell death by combinations of SAHA and PLX4720 is largely independent of the caspase cascade. (**a**) MM200, Sk-Mel-28, IgR3, and Mel-RMu cells with or without pretreatment with z-VAD-fmk (30 *μ*M) for 1 h were treated with SAHA (2 *μ*M), PLX4720 (5 *μ*M), or the combination of both for a further 48 h. MM200 and Mel-RMu cells treated with TNF-related apoptosis-inducing ligand (TRAIL) (200 ng/ml) with or without pretreatment with z-VAD-fmk were included as controls. Cell viability was measured by CellTiter-Glo assays. The data shown are mean±S.E.M. of three individual experiments. *P<0.01, two-tailed Student's *t*-test. (**b**) MM200, Sk-Mel-28, IgR3, and Mel-RMu cells were transfected with the control or caspase-3 siRNA. After 24 h, whole-cell lysates were subjected to western blot analysis of caspase-3 and glyceraldehyde 3-phosphate dehydrogenase (GAPDH) (as a loading control). The data shown are representative of three individual experiments. (**c**) MM200, Sk-Mel-28, IgR3, and Mel-RMu cells were transfected with the control or caspase-3 siRNA. After 24 h, cells were treated with SAHA (2 *μ*M), PLX4720 (5 *μ*M), or the combination of both for a further 48 h. Cell viability was measured by CellTiter-Glo assays. The data shown are mean±S.E.M. of three individual experiments. *P<0.01, two-tailed Student's *t*-test

Because synergistic killing of BRAF^V600E^ melanoma cells by SAHA and PLX4720 was associated with the activation of caspase-3 and -9 (Figure 1d), we reasoned that the caspase cascade had an important role in enhanced induction of cell death. However, the general caspase inhibitor Z-Val-Ala-Asp(OMe)-CH_2_F (z-VAD-fmk) did not inhibit melanoma cell death induced by the combination, while it efficiently blocked killing by TNF-related apoptosis-inducing ligand in sensitive MM200 and Mel-RMu cells (Figure 3a).^40^ Similarly, z-VAD-fmk had only a negligible inhibitory effect on cell death induced by PLX4720 alone in sensitive Mel-RMu cells (Figure 3a), in line with caspase-independent killing of melanoma cells by the MEK inhibitor U0126.^21^ On the other hand, z-VAD-fmk significantly inhibited cell death induced by SAHA plus PLX4720 or by SAHA alone in IgR3 cells (*P*<0.05, two-tailed Student's *t*-test) (Figure 3a). These results suggest that the combination of SAHA and PLX4720 can bypass the caspase cascade in a cell line-dependent manner to kill BRAF^V600E^ melanoma cells. This was further consolidated in experiments with caspase-3, the major effector caspase, knocked down by siRNA (Figures 3b and c).

### Cotreatment with SAHA and PLX4720 triggers necrosis in BRAF^V600E^ melanoma cells

**Figure 4:**
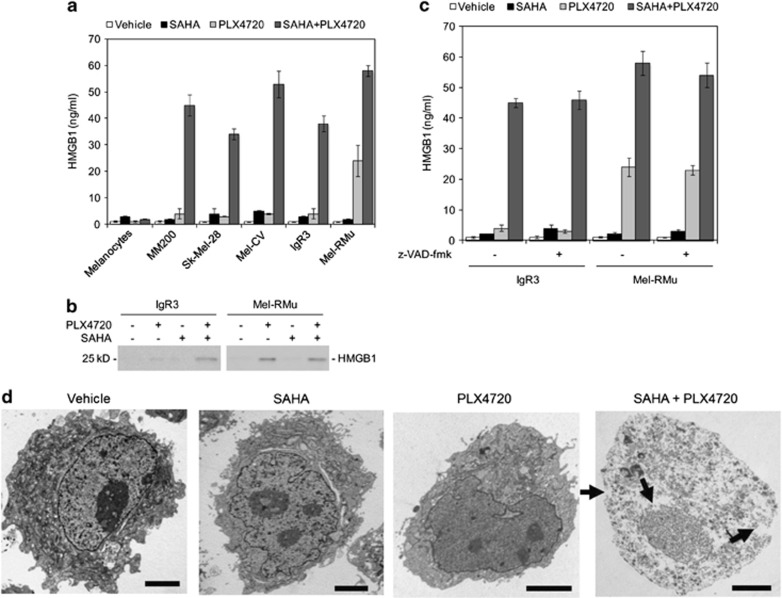
Cotreatment with SAHA and PLX4720 triggers necrosis in BRAF^V600E^ melanoma cells. (**a**) HEMn-MP melanocytes, MM200, Sk-Mel-28, Mel-CV, IgR3, and Mel-RMu melanoma cells were treated with SAHA (2 *μ*M), PLX4720 (5 *μ*M), or the combination of both for 24 h. HMGB1 in the cultured medium was quantitated by ELISA. The data shown are the mean±S.E.M. of three individual experiments with triplicate assays in each experiment. (**b**) IgR3 and Mel-RMu cells were treated with SAHA (2 *μ*M), PLX4720 (5 *μ*M), or the combination of both for 24 h. Proteins in the culture medium were concentrated using centrifugal filter units with the Ultracel membrane. Twenty microliters of the resultants were subjected to western blot analysis of HMGB1. The data shown are representative of three individual experiments. (**c**) IgR3 and Mel-RMu cells pre-treated with z-VAD-fmk (30 *μ*M) for 1 h were treated with SAHA (2 *μ*M), PLX4720 (5 *μ*M), or the combination of both for a further 24 h. HMGB1 in the cultured medium was quantitated by ELISA. The data shown are the mean±S.E.M. of three individual experiments with triplicate assays in each experiment. (**d**) MM200 cells treated with the vehicle (DMSO), SAHA (2 *μ*M), PLX4720 (5 *μ*M), or the combination of SAHA and PLX4720 were subjected to transmission electron microscopic analysis. Representative micrographs showing that cotreatment with SAHA and PLX4720 induced rupture of the nuclear and cell membrane (arrowheads), and loss of nuclear and cytoplasmic contents, features of necrosis. Scale bar: 2.5 *μ*m ( × 4000)

To clarify the mode of BRAF^V600E^ melanoma cell death induced by the combination of SAHA and PLX4720, we monitored release of the intracellular protein high-mobility group protein B1 (HMGB1) in relation to activation of the caspase cascade. The release of HMGB1 was readily detectable in BRAF^V600E^ melanoma cells cotreated with SAHA and PLX4720, which appeared caspase-independent, as z-VAD-fmk did not alter the levels of extracellular HMGB1 (Figures 4a–c), indicating that the release is not secondary to apoptosis.^41^ These results, along with caspase-independent induction of cell death and the observation that melanoma cells instantly became positive for PI along with Annexin V when committing to death, suggest that the combination of SAHA and PLX4720 may primarily induce necrosis in melanoma cells (Figures 1b and 3).^32, 33^ Notably, PLX4720 alone triggered caspase-independent release of HMGB1 in sensitive Mel-RMu cells (Figures 4a–c). In contrast, SAHA did not cause HMGB1 release even in sensitive IgR3 cells (Figures 4a and b).

To confirm the mode of cell death induced by SAHA in combination with PLX4720 in BRAF^V600E^ melanoma cells, we performed transmission electron microscopic analysis. Necrotic cell death manifested by rupture of the plasma membrane and loss of nuclear and cytoplasmic contents was readily detected using transmission electron microscopy in MM200 cells cotreated with SAHA and PLX4720 (Figure 4d). In contrast, MM200 cells treated with SAHA or PLX4720 alone resembled those treated with the vehicle control (dimethyl sulfoxide (DMSO)), displaying intact plasma membrane and preserved nuclear architecture (Figure 4d). Nuclear fragmentation was uncommon in cells treated with SAHA, PLX4720, or SAHA plus PLX4720. Thus, the combination of SAHA and PLX4720 primarily induces necrosis in BRAF^V600E^ melanoma cells.

### Neither RIPK1 nor RIPK3 is required for synergistic killing of BRAF^V600E^ melanoma cells by SAHA and PLX4720

**Figure 5:**
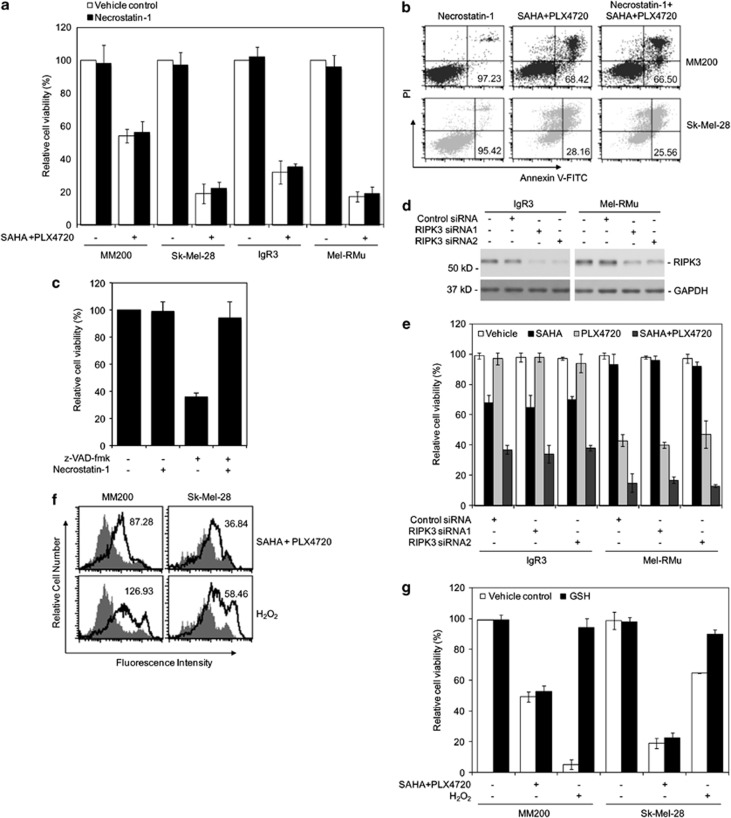
RIPK1, RIPK3, and ROS are not involved in killing of BRAF^V600E^ melanoma cells by combinations of SAHA and PLX4720. (**a**) MM200, Sk-Mel-28, IgR3, and Mel-RMu cells with or without pretreatment with nec-1 (30 *μ*M) for 1 h were treated with the combination of SAHA (2 *μ*M) and PLX4720 (5 *μ*M) for a further 48 h. Cell viability was measured by CellTiter-Glo assays. The data shown are mean±S.E.M. of three individual experiments. (**b**) MM200 and Sk-Mel-28 cells with or without pretreatment with nec-1 (30 *μ*M) for 1 h were treated with the combination of SAHA (2 *μ*M) and PLX4720 (5 *μ*M) for a further 48 h. Induction of cell death was quantitated by the Annexin V-fluorescein isothiocyante (FITC)/propidium iodide (PI) method. The number in each right bottom quadrant represents the percentage of viable cells in each sample. The data shown are representative of three individual experiments. (**c**) L929 cells with or without pretreatment with nec-1 (30 *μ*M) for 1 h were treated with z-VAD-fmk (20  *μ*M) for 24 h. Cell viability was measured by CellTiter-Glo assays. The data shown are mean±S.E.M. of three individual experiments. (**d**) IgR3 and Mel-RMu cells were transfected with the control or RIPK3 siRNA. After 24 h, whole-cell lysates were subjected to western blot analysis of RIPK3 and glyceraldehyde 3-phosphate dehydrogenase (GAPDH) (as a loading control). The data shown are representative of three individual experiments. (**e**) IgR3 and Mel-RMu cells were transfected with the control or RIPK3 siRNA. After 24 h, cells were treated with SAHA (2  *μ*M), PLX4720 (5  *μ*M), or the combination of both for a further 48 h. Cell viability was measured by CellTiter-Glo assays. The data shown are mean±S.E.M. of three individual experiments. (**f**) Representative flow cytometry histograms of assays of ROS production. MM200 and Sk-Mel-28 cells were treated with the vehicle (DMSO) (filled histograms) or the combination of SAHA (2 *μ*M) and PLX4720 (5 *μ*M) (open histograms) for 36 h. Cells treated with hydrogen peroxide (open histograms) were included as controls. The numbers represent mean fluorescence intensity (MFI) of each testing samples relative to their controls. (**g**) MM200 and Sk-Mel-28 cells were treated with the antioxidant GSH (10 *μ*M) for 2 h before adding SAHA (2 *μ*M) and PLX4720 (5 *μ*M) for another 48 h. Cell viability was measured by CellTiter-Glo assays. The data shown are mean±S.E.M. of three individual experiments. *P<0.01, two-tailed Student's *t*-test

As RIPK1 has an important role in initiating programmed necrosis in many types of cells induced by a variety of stimuli,^32, 33^ we examined whether it is involved in necrosis of melanoma cells induced by cotreatment with SAHA and PLX4720. To this end, we treated MM200, Sk-Mel-28, IgR3, and Mel-RMu cells with necrostatin-1 (Nec-1), which blocks necrotic signaling by inhibiting RIPK1,^42, 43^ 1 h before the addition of SAHA and PLX4720. As shown in Figures 5a and b, Nec-1 did not inhibit melanoma cell death induced by SAHA and PLX4720, nor did it inhibit cell death induced by PLX4720 alone in Mel-RMu and cell death induced by SAHA alone in IgR3 cells (data not shown). As expected, Nec-1 efficiently blocked necrosis (necroptosis) induced by z-VAD-fmk in L929 cells that were used as a control (Figure 5c).^44, 45^

We also examined whether RIPK3, which can mediate necrotic signaling dependently or independently of RIPK1,^46^ contributes to induction of necrosis by SAHA and PLX4720. Similar to inhibition of RIPK1, siRNA knockdown of RIPK3 had no effect on killing of IgR3 and Mel-RMu cells by cotreatment with SAHA and PLX4720, nor did it affect Mel-RMu cell death induced by PLX4720 and IgR3 cell death induced by SAHA (Figures 5d and e). Collectively, these results indicate that the combination of SAHA and PLX4720 induces necrosis of melanoma cells independently of RIPK1 and RIPK3.

As induction of necrosis commonly involves generation of reactive oxygen species (ROS),^47^ we examined if ROS production is increased by cotreatment with SAHA and PLX4720. Figure 5f shows that the levels of ROS were increased, albeit moderately, in MM200 and Sk-Mel-28 cells treated with the combination of the inhibitors. However, the antioxidant glutathione (GSH) did not impinge on cell death induced by SAHA and PLX4720, but markedly inhibited cell killing by hydrogen peroxide that was used as a control (Figure 5g), indicating that the generation of ROS does not have a major role in induction of necrosis by cotreatment with SAHA and PLX4720.

### SAHA and vemurafenib cooperatively inhibits BRAF^V600E^ melanoma growth in a xenograft mouse model

**Figure 6:**
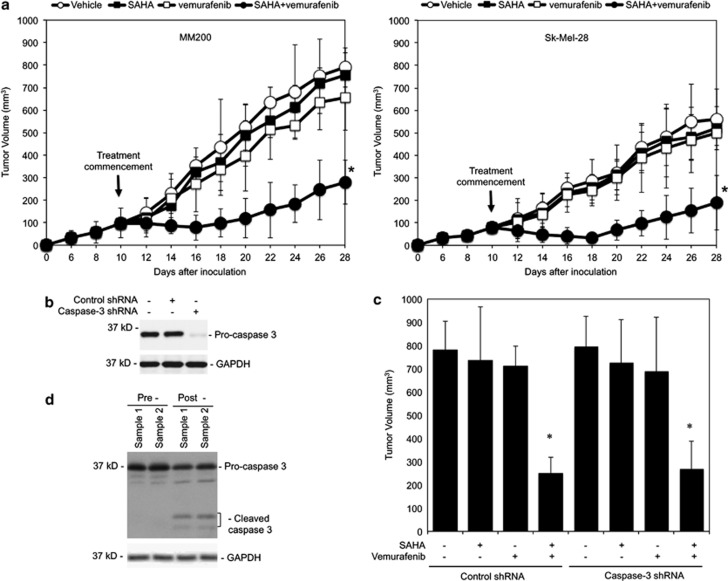
Cotreatment with SAHA and vemurafenib inhibits melanoma xenograft growth in a mouse model. (**a**) MM200 (left) and Sk-Mel-28 (right) cells (1 × 10^7^) were xenografted into flanks of nu/nu mice viasubcutaneous injection. Ten days after transplantation when xenografts reached approximately 100 mm^3^, mice were administered with either the vehicle (DMSO) (*n*=8) or SAHA (100 mg/kg per day) (*n*=8) via intraperitoneal injection, vemurafenib (75 mg/kg per day) (*n*=8) via oral gavage, or the combination of SAHA and vemurafenib daily for 10 days. Mice were euthanized at 28 days after melanoma cell injection. The data shown are growth curves of melanoma tumors represented by the volume calculated with the modified ellipsoidal formula (tumor volume=1/2(length × width^2^)), which are mean±S.E.M. of all tumors in each experimental group (*P<0.001, Student's *t*-test). (**b**) Whole-cell lysates from MM200 cells transduced with the control or caspase-3 short hairpin RNA (shRNA) were subjected to western blot analysis of caspase-3 and glyceraldehyde 3-phosphate dehydrogenase (GAPDH) (as a loading control). The data shown are representative of three individual western blots. (**c**) MM200 cells transduced with the control or caspase-3 shRNA were xenografted into flanks of nu/nu mice via subutaneous injection. Ten days after transplantation, mice were administered with either the vehicles (*n*=8) or SAHA (100 mg/kg per day) (*n*=8) via intraperitoneal injections, vemurafenib (75 mg/kg per day) (*n*=8) via oral gavage, or the combination of SAHA and vemurafenib daily for 10 days. Mice were euthanized at 28 days after melanoma cell injection. The data shown are tumor volume at the date of euthanization, which are mean±S.E.M. of all tumors in each experimental group (*P<0.001, Student's *t*-test). (**d**) Whole-cell lysates of crude tumor tissues randomly sampled from tumors formed with MM200 cells transduced with the control shRNA before and undergoing treatment with SAHA in combination with vemurafenib were subjected to western blot analysis of caspase-3 and GAPDH (as a loading control). The data shown are representative of three individual experiments

To examine the combinatorial effect of HDAC and mutant BRAF inhibitors on melanoma cells *in vivo*, we transplanted subcutaneously MM200 and Sk-Mel-28 cells, which were resistant to PLX4720 or SAHA alone *in vitro* (Figure 1a),^36^ into nu/nu mice. Mice carrying established xenografts were treated with vehicle, SAHA, vemurafenib, or SAHA plus vemurafenib. As shown in Figure 6a, neither vemurafenib nor SAHA significantly impinged on growth of MM200 and Sk-Mel-28 xenografts (*P*>0.05, two-tailed Student's *t*-test), consistent with resistance of the cells to PLX4720 or SAHA *in vitro* (Figure 1a).^36^ However, cotreatment with the inhibitors markedly inhibited tumor growth (*P*<0.001, two-tailed Student's *t*-test) (Figure 6a). Of note, cotreatment did not cause significant changes in body weights or physical abnormality of the mice, suggesting that it is tolerable *in vivo*.

We also examined the xenografts of MM200 cells with caspase-3 knocked down by shRNA to test whether inhibition of melanoma growth by the combination of SAHA and vemurafenib *in vivo* is similarly caspase-independent. Figures 6b and c show that cotreatment with SAHA and vemurafenib inhibited tumor growth to similar extents in xenografts deficient in caspase-3 and those carrying control shRNA, although caspase-3 was activated in the latter as shown by the analysis of xenograft samples harvested during treatment (Figure 6d).

## Discussion

The above results extend our previous finding that HDAC and BRAF inhibitors synergistically induce cell death of BRAF^V600E^ melanoma cells by showing that, although the combination triggers activation of the caspase cascade and the mitochondrial apoptotic signaling, it kills BRAF^V600E^ melanoma cells primarily by induction of necrosis through a mechanism that is independent of RIPK1 and RIPK3. In addition, the results reveal that coadministration of the HDAC inhibitor SAHA and the BRAF inhibitor vemurafenib inhibits melanoma xenograft growth independently of caspases *in vivo*. Therefore, cotargeting HDACs and mutant BRAF can bypass canonical cell death pathways to kill BRAF^V600E^ melanoma cells. This may be therapeutically beneficial, in that melanoma cells have commonly developed resistance mechanisms against conventional cell death signaling.^48^

Apoptosis has been widely documented to be responsible for cell death induced by BRAF and MEK inhibitors.^3, 4, 17^ However, our results in this study suggest that programmed necrosis is the major mode of cell death in BRAF^V600E^ melanoma cells induced by the combination of SAHA and PLX4720. This was directly evidenced by visualization of rupture of the plasma membrane and loss of nuclear and cytoplasmic contents using transmission electron microscopy. The absence of nuclear fragmentation argues against necrosis secondary to apoptosis. Moreover, induction of necrosis was also indirectly supported by a number of findings. These include (1) cell killing by the combination was largely caspase-independent; (2) uptake of PI was an early event when cells committed to death; and (3) caspase-independent release of HMGB1.^32, 49^ Nevertheless, induction of cell death was associated with activation of the caspase cascade and mitochondrial apoptotic signaling and cleavage of PARP into a 89 kDa fragment, indicating that the caspase-dependent, mitochondrion-mediated apoptotic machinery was also activated.^38, 39^ We have previously reported that the MEK inhibitor U0126 induces caspase-independent apoptosis in the face of activation of the caspase cascade in melanoma cells.^21^ SAHA can also induce caspase-independent cell death in many types of cells including Sk-Mel-28 melanoma cells.^30, 31, 50^ It is conceivable that, in addition to necrosis, caspase-independent apoptosis may also contribute to cell death induced by the combination of SAHA and PLX4720 in BRAF^V600E^ melanoma cells.

Induction of programmed necrosis is emerging as an important mechanism to kill cells under various cellular stresses.^32, 33^ Although mechanisms involved remain to be fully characterized, RIPK1- and RIPK3-mediated signaling is responsible for necrosis induced by the activation of death receptors and many other stimuli such as DNA-damaging drugs.^33, 44, 51^ As such, nec-1 that was initially identified as an allosteric inhibitor of RIPK1 has been commonly used as a tool for inhibition of necrosis.^34, 42, 43, 45, 52^ Although it is now known that Nec-1 is identical to methyl-thiohydantoin-tryptophan that also inhibits the immunomodulator indoleamine-2,3-dioxygenase,^42, 45^ its inhibitory effect on necrosis is due to its ability to inhibit RIPK1.^45^ Nec-1 did not inhibit cell death induced by cotreatment with SAHA and PLX4720, whereas it markedly blocked cell death (necroptosis) induced by the caspase inhibitor z-VAD-fmk in L929 cells that were used as a positive control.^44, 45^ Likewise, siRNA knockdown of RIPK3 did not impact on cell death induced by cotreatment with SAHA and PLX4720. These results indicate that neither RIPK1 nor RIPK3 is required for killing of BRAF^V600E^ melanoma cells by combinations of HDAC and BRAF inhibitors. RIPK1- and RIPK3-independent induction of necrosis has been reported in other experimental systems.^53, 54, 55^

Induction of programmed necrosis has recently been shown to involve sequential activation of MLKL, PGAM5, and Drp1 downstream of RIPK1 and RIPK3.^34, 35^ We attempted to examine the role of involvement of MLKL and Drp1 in BRAF^V600E^ melanoma cell death induced by cotreatment with SAHA and PLX4720 using the commercially available inhibitors necrosulfonamide and mdivi-1, respectively.^34, 35^ However, these inhibitors displayed extensive toxicity towards melanoma cells even when used at concentrations 5- to 10-fold lower than previously reported (data not shown).^34, 35^ These observations suggest that MLKL and Drp1 may have more profound roles in regulating melanoma cell survival, but whether they are involved in necrosis induced by combinations of HDAC and BRAF inhibitors remains to be clarified.

Another mechanism that is commonly involved in induction of necrosis is generation of ROS.^47^ Indeed, HDAC inhibitors can kill cells by the production of ROS independently of caspase activation.^56, 57^ However, although ROS were produced in BRAF^V600E^ melanoma cells by treatment with SAHA in combination with PLX4720, they did not appear to be involved in induction of necrosis as the antioxidant GSH was unable to prevent the cells from death. Intriguingly, the combination induced an increase in a ∼50 kDa fragment detected by an antibody against PARP that corresponded to a band generated by necrotic cleavage of PARP by cathepsins,^38, 39^ suggesting that cathepsins may have a role in necrosis of melanoma cells cotreated with the inhibitors. However, this band was also detectable in untreated melanoma cells at markedly higher levels than the native form of PARP. Whether PARP is constitutively cleaved in melanoma cells by proteases such as cathepsins in the absence of cell death warrants further investigations.^38, 39^

Although we and others have previously found that upregulation of Bim is important for killing of sensitive melanoma cells by inhibition of the MEK/ERK pathway,^10, 17, 21^ our results in this study showed that involvement of Bim is, at least in some BRAF^V600E^ melanoma cell lines, dispensable for induction of cell death by cotreatment with SAHA and PLX4720. Nonetheless, overexpression of Mcl-1 inhibited, albeit partially, cell death regardless of whether Bim is involved, suggesting that combinations of HDAC and BRAF inhibitors can exert damage to the mitochondria, which is important in regulating both apoptosis and necrosis, by mechanisms alternative to activation of Bim.^33, 34, 35^ Antiapoptotic Bcl-2 family proteins such as Bcl-X_L_ is known to bind to pronecrosis proteins including PGAM5 and Drp1 in addition to interactions with proapoptotic proteins.^58^ Whether other prosurvival Bcl-2 family proteins such as Mcl-1 can similarly do so remains unknown. In this regard, it is worth noting that the BH3-only protein Bmf has recently been implicated in induction of necrosis.^35^

In summary, we have shown in this report that combinations of HDAC and BRAF inhibitors synergistically kill BRAF^V600E^ melanoma cells by induction of necrosis. Although the exact mechanism by which the two classes of inhibitors interact to induce necrosis of BRAF^V600E^ melanoma cells remains to be defined, a number of factors including RIPK1, RIPK3, and generation of ROS do not appear to have a major role. Regardless, the ability to bypass canonical cell death pathways to kill melanoma cells by combinations of HDAC and BRAF inhibitors may be of therapeutic advantage. In support, coadministration of SAHA and vemurafenib cooperatively inhibits melanoma xenograft growth *in vivo* in a caspase-independent manner.

## Materials and Methods

### Cell lines, antibodies, and other reagents

Human melanoma cell lines MM200, Sk-Mel-28, Mel-CV, IgR3, and Mel-RMu have been described previously.^17, 29^ The murine fibrosarcoma cell line L929 was purchased from Sigma-Aldrich (St. Louis, MO, USA). All cell lines were cultured in Dulbecco's modified Eagle's medium (DMEM) containing 5% fetal calf serum (FCS) (Commonwealth Serum Laboratories, Parkville, VIC, Australia). The human melanocyte cell line HEMn-MP was purchased from Banksia Scientific (Bulimba, QLD, Australia) and cultured in melanocyte medium (Gibco, Invitrogen, Mulgrave, VIC, Australia). The mouse monoclonal antibodies (mAbs) against phospho-ERK1/2 (Thr202/Tyr204) and Mcl-1 and rabbit polyclonal (pAb) against Smac/DIABLO were from Santa Cruz Biotechnology (Santa Cruz, CA, USA); the mouse mAbs against COX IV and rabbit pAb against cytochrome *C* were from Clontech (Mountain View, CA, USA); the rabbit pAb against ERK1/2 was from Cell Signaling Technology (Beverly, MA, USA); the rabbit pAb against Bim was from Imgenex (San Diego, CA, USA); the rabbit pAbs against caspase-3 and caspase-9 were from Enzo Life Sciences (Farmingdale, NY, USA); the rabbit pAbs against *β*-actin, HMGB1, and RIPK3 were from Abcam (Cambridge, MA, USA); the mouse mAb against PARP was from BD Pharmingen (Bioclone, Marrickville, NSW, Australia); the rabbit pAb against PARP p85 fragment was from Promega (San Luis Obispo, CA, USA); and the mouse mAb against GAPDH was from Ambion (Austin, TX, USA). PLX4720 was provided by Plexxikon Inc. (Berkeley, CA, USA). It was dissolved in DMSO and made up in stock solutions of 4 mM. SAHA and LBH589 was purchased from Selleck (Burwood East, VIC, Australia), which were dissolved in DMSO and made up in stock solutions of 20 mM and 70 mg/ml, respectively. The cell-permeable general caspase inhibitor z-VAD-fmk was purchased from Calbiochem (La Jolla, CA, USA). Nec-1 was purchased from Sigma-Aldrich Pty Ltd (Sydney, NSW, Australia).

### CellTiter-Glo assay

The CellTiter-Glo assay was performed with the CellTiter-Glo Luminescent Cell Viability Assay kit according to the manufacturer's instructions (Promega, San Luis Obispo, CA, USA). Luminescence was recorded by Synergy 2 multidetection microplate reader (Biotek, Winooski, VT, USA).

### Annexin V and PI staining

Staining with PI- and FITC-conjugated Annexin V was carried out according to the manufacturer's instructions and as described elsewhere.^29^ In brief, 1 × 10^6^ cells per sample were collected, washed two times with cold PBS, and re-suspended in 1 × Annexin V binding buffer. Cells were incubated in 1% Annexin V-FITC and PI for 15 min in the dark, an additional 400 *μ*l of binding buffer was added to each tube, and cells were analyzed by flow cytometry within 1 h.

### Measurement of mitochondrial membrane potential

Melanoma cells were seeded at 1 × 10^5^ cells per well in 24-well plates and allowed to reach exponential growth for 24 h before treatment. Changes in mitochondrial membrane potential (ΔΨ_m_) were studied by staining the cells with the cationic dye, JC-1, according to the manufacturer's instructions (Molecular Probes, Eugene, OR USA) as described previously.^20, 21^

### Mitochondrial and cytosolic fractions

Methods used for subcellular fractionation were similar to those described previously.^21, 29^ Cell pellets were then suspended in five volumes of buffer A (20 mM HEPES–KOH (pH 7.5), 10 mM KCI, 1 mM Na-EGTA, 1 mM DTT, and 0.1 mM phenylmethylsulfonyl fluoride containing 250 mM sucrose) supplemented with protease inhibitor cocktail tablets. After incubation on ice for 15 min, the cells were disrupted by passing them 15 times through a 22-G needle. After centrifugation two times at 750 × *g* for 10 min at 4 °C, the supernatant was collected and centrifuged at 10 000 × *g* for 15 min at 4 °C, and the resulting mitochondrial pellets were resuspended in buffer A. The supernatants of the 10 000 spin were further centrifuged at 100 000 × *g* for 1 h at 4 °C, and the resulting supernatants were designated as the S-100 cytosolic fraction.

### Clonogenic assays

Clonogenic assays were performed as described previously.^10^ Briefly, cells were seeded at 1000 cells per well onto 6-well culture plates and allowed to grow for 24 h, followed by the desired treatment. At 48 h after the addition of respective drugs, the culture medium was changed to fresh DMEM containing 5% FCS, where cells were then allowed to grow for a further 12 days before fixation with methanol and staining with 0.5% crystal violet. The images were captured with Bio-Rad VersaDoc image system (Bio-Rad, Gladesville, NSW, Australia).

### Measurement of extracellular HMGB1

Quantitation of extracellular HMGB1 in the culture medium by enzyme-linked immunosorbent assay (ELISA) was performed as described previously.^59^ Briefly, 10 μl of standard, positive control, and conditioned medium were added to a microtiter plate containing the diluent buffer provided by in the HMGB1 ELISA kit (IBL, Hamburg, Germany), followed by overnight incubation at 37 °C in the dark. The plate was then washed and incubated with the enzyme conjugate for 2 h, followed by the addition of the color solution for 30 min. Stop solution was added before measurement of optical density by Synergy 2 multidetection microplate reader (Biotek, Winooski, VT, USA).

To quantitate extracellular HMGB1 in the culture medium by the western blotting, supernatant from the conditioned medium was firstly condensed using the Amicon Ultra-0.5 Centrifugal Filter Unit (Merck Millipore, Kilsyth, VIC, Australia) according to the manufacturer's instructions. The condensed proteins were then quantitated and subjected to western blot analysis.

### Measurement of ROS generation

Generation of ROS was monitored by measurement of hydrogen peroxide generation. Cells that were seeded in 24-well plates overnight with or without treatment with vehicle control (DMSO), SAHA plus PLX4720, or H_2_O_2_ (positive control) were incubated with the fluorescent probe 2′,7-dichlorofluorescein diacetate (DCF-DA; Sigma Chemical, St Louis, MO, USA) for 30 min. The medium was removed to a 75-mm Falcon polystyrene tube and the adherent cells were trypsinized and collected into the same tube. After washing two times with PBS, the intensity of DCF-DA fluorescence was determined by using a FACScan flow cytometer (Becton Dickinson, Sunnyvale, CA, USA), with an excitation wavelength of 480 nm and an emission wavelength of 530 nm.

### Transmission electron microscopy

Transmission electron microscopy was used to analyze cell morphology and intracellular structure to determine the type of cell death in melanoma cell lines. Cells were harvested, chemically fixed in 2.5% glutaraldehyde and 2% paraformaldehyde in 0.1 M sodium phosphate buffer (pH 7.2), washed and then embedded in molten 4% agarose gel. Trimmed agar blocks containing fixed cells were subsequently fixed in 1% osmium tetroxide. *En bloc* staining of samples was carried out by submerging agar blocks in 2% uranyl acetate. Agar blocks were then rinsed in water and dehydrated. Next, resin infiltration was performed by submerging blocks in increasing gradients of ethanol and Procure Resin, followed by embedding in pure Procure Resin. Samples in resin were then polymerized by incubating them at 60°C for 24 h. Polymerized resin blocks were then cut to 70-nm-thick sections with Leica ultramicrotome. Sections were mounted onto Formvar non-carbon-coated grids and positively stained with 2% uranyl acetate and lead citrate solution. Stained samples on grids were visualized using a JEOL 1400 TEM and digital micrographs of individual cells were acquired at × 4000 magnification with Gatan Digital Micrograph software (Pleasanton, CA, USA).

### Western blot analysis

Western blot analysis was carried out as described previously.^10, 60^ Labeled bands were detected by Luminata Crescendo Western HRP substrate (Millipore, Billerica, MA, USA) and images were captured and the intensity of the bands was quantitated with ImageReader LAS-4000 (Fujifilm Corporation, Tokyo, Japan).

### Plasmid vector and transfection

Mcl-1 cDNA cloned into p3 × FLAG-cytomegalovirus-10 was provided by Dr. Xiaodong Wang (Howard Hughes Medical Institute, Dallas, TX, USA) and described elsewhere.^60^ Cells were transfected with 2 *μ*g plasmid as well as the empty vector in Opti-MEM medium (Invitrogen, Carlsbad, CA, USA) with Lipofectamine 2000 reagent (Invitrogen) according to the manufacturer's protocol. At 6 h after transfection, the cells were switched into antibiotic-free medium containing 5% FCS for a further 24 h. Cells were then passaged at 1 : 10 ratio into the fresh medium for further 24 h, followed by G418 (Sigma-Aldrich) selection.

### Small interference RNA

The siRNA constructs used were obtained as the siGENOME SMARTpool reagents (Dharmacon, Lafayette, CO, USA). The siRNA constructs used were: Bim siGENOME SMARTpool (M-004383-01-0010), caspase-3 siGENOME SMARTpool (M-004307-02-0010), and non-targeting siRNA pool (D-001206-13-20) as control. RIPK3-homo-350 (5′-GCGGUCAAGAUCGUAAACUTT-3′), RIPK3-homo-1548 (5′-GACCGCUCGUUAACAUAUATT-3′) and non-targeting (5′-UUCUCCGAACGUGUCACGUTT-3′) siRNAs were obtained from Shanghai GenePharma Co. Ltd (Zhangjiang Hi-Tech Park, Shanghai, P.R. China). Transfection of siRNA pools was carried out as described previously.^13, 60^

### Xenograft experiments

Melanoma cells (1 × 10^7^) were subcutaneously injected into each flank of male athymic nude mice (Model Animal Research Centre of Nanjing University, Nanjing, China). Ten days after injection, when xenografts were approximately 100 mm^3^, mice were randomly assigned into different groups. Mice were treated daily with SAHA (100 mg/kg per day in sterile PBS via intraperitoneal injection) (*n*=8), vemurafenib (75 mg/kg per day in PBS via oral gavage) (*n*=8), SAHA plus vemurafenib (*n*=8), or equivalent volumes of vehicles (*n*=8) for 10 days. Mouse weights and tumor volumes (1/2(length × width^2^) were measured three times per week. Mice were killed at 28 days after tumor cell transplantation. Studies on animals were approved by the Animal Research Ethics Committee of Shanxi Cancer Hospital (Shanxi, China).

### Statistical analysis

The significance of differences between experimental data was determined using the two-tailed Student's *t*-test for unpaired observations. *P*<0.05 was considered to be statistically significant. The Fa–CI plot method for constant ratio combinations, derived from the median-effect principle of Chou and Talalay, was used to analyze quantitatively the interaction between SAHA and PLX4720 using the commercially available software CalcuSyn (Biosoft, Cambridge, UK; www.biosoft.com).

## Abbreviations

HDAC: histone deacetylase
BRAF: v-Raf murine sarcoma viral oncogene homolog B1
ERK: extracellular signal-regulated kinases
SAHA: suberoylanilide hydroxamic acid
LBH589: panobinostat
RIPK: receptor-interacting protein kinase
HMGB1: high-mobility group protein B1
