# Supplementary material for: Cotargeting histone deacetylases and oncogenic BRAF synergistically kills human melanoma cells by necrosis independently of RIPK1 and RIPK3
Source: Cell Death Dis. 2013 Jun 6;4(6):e655–. doi: 10.1038/cddis.2013.192 (PMC3702278; doi:10.1038/cddis.2013.192)
Supplement: Supplementary Figures [file cddis2013192x1.ppt]

## Slide 1
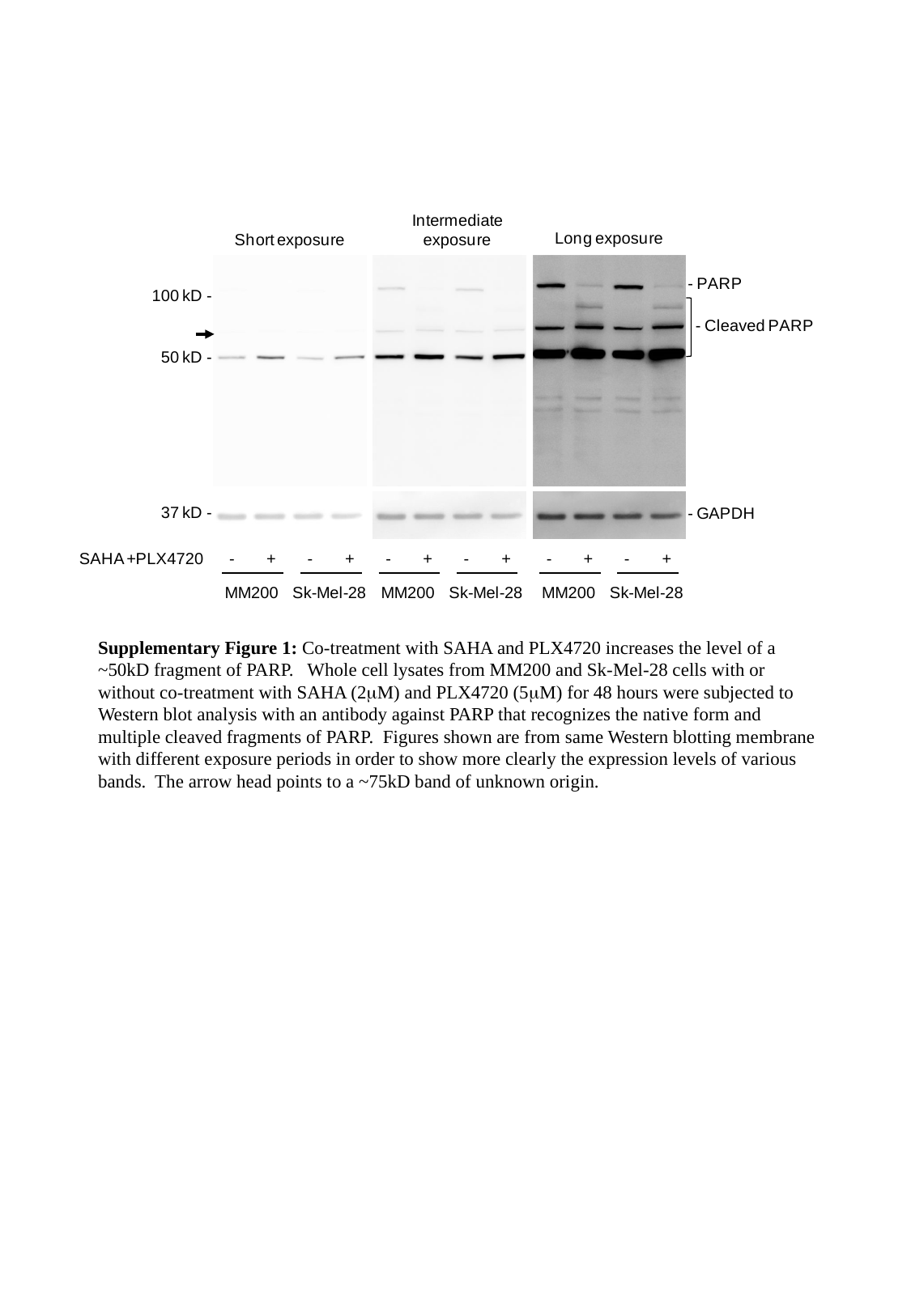

Supplementary Figure 1: Co-treatment with SAHA and PLX4720 increases the level of a ~50kD fragment of PARP. Whole cell lysates from MM200 and Sk-Mel-28 cells with or without co-treatment with SAHA (2μM) and PLX4720 (5μM) for 48 hours were subjected to Western blot analysis with an antibody against PARP that recognizes the native form and multiple cleaved fragments of PARP. Figures shown are from same Western blotting membrane with different exposure periods in order to show more clearly the expression levels of various bands. The arrow head points to a ~75kD band of unknown origin.

## Slide 2
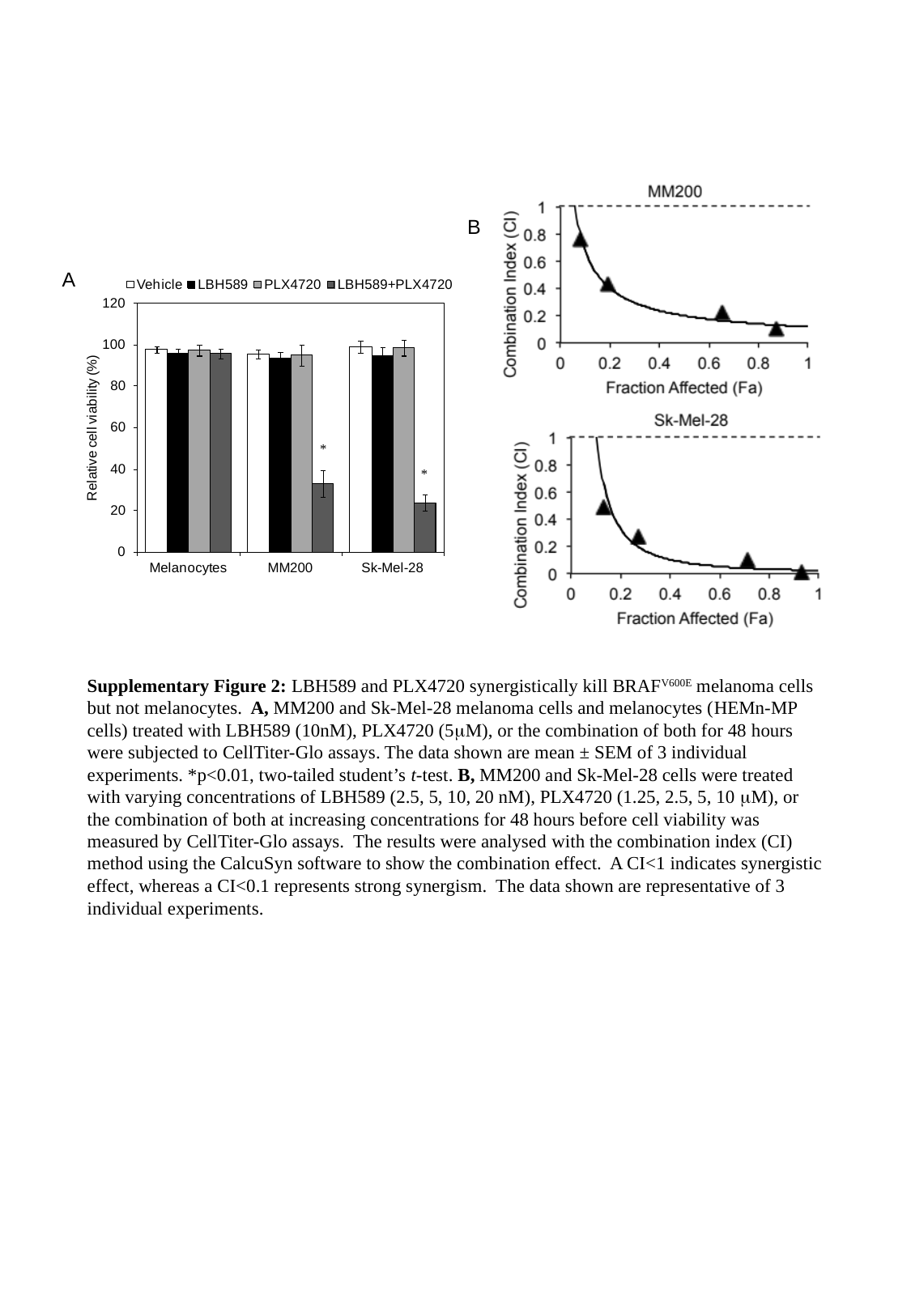

B
A
*
*
Supplementary Figure 2: LBH589 and PLX4720 synergistically kill BRAFV600E melanoma cells but not melanocytes. A, MM200 and Sk-Mel-28 melanoma cells and melanocytes (HEMn-MP cells) treated with LBH589 (10nM), PLX4720 (5μM), or the combination of both for 48 hours were subjected to CellTiter-Glo assays. The data shown are mean ± SEM of 3 individual experiments. *p<0.01, two-tailed student’s t-test. B, MM200 and Sk-Mel-28 cells were treated with varying concentrations of LBH589 (2.5, 5, 10, 20 nM), PLX4720 (1.25, 2.5, 5, 10 μM), or the combination of both at increasing concentrations for 48 hours before cell viability was measured by CellTiter-Glo assays. The results were analysed with the combination index (CI) method using the CalcuSyn software to show the combination effect. A CI<1 indicates synergistic effect, whereas a CI<0.1 represents strong synergism. The data shown are representative of 3 individual experiments.

## Slide 3
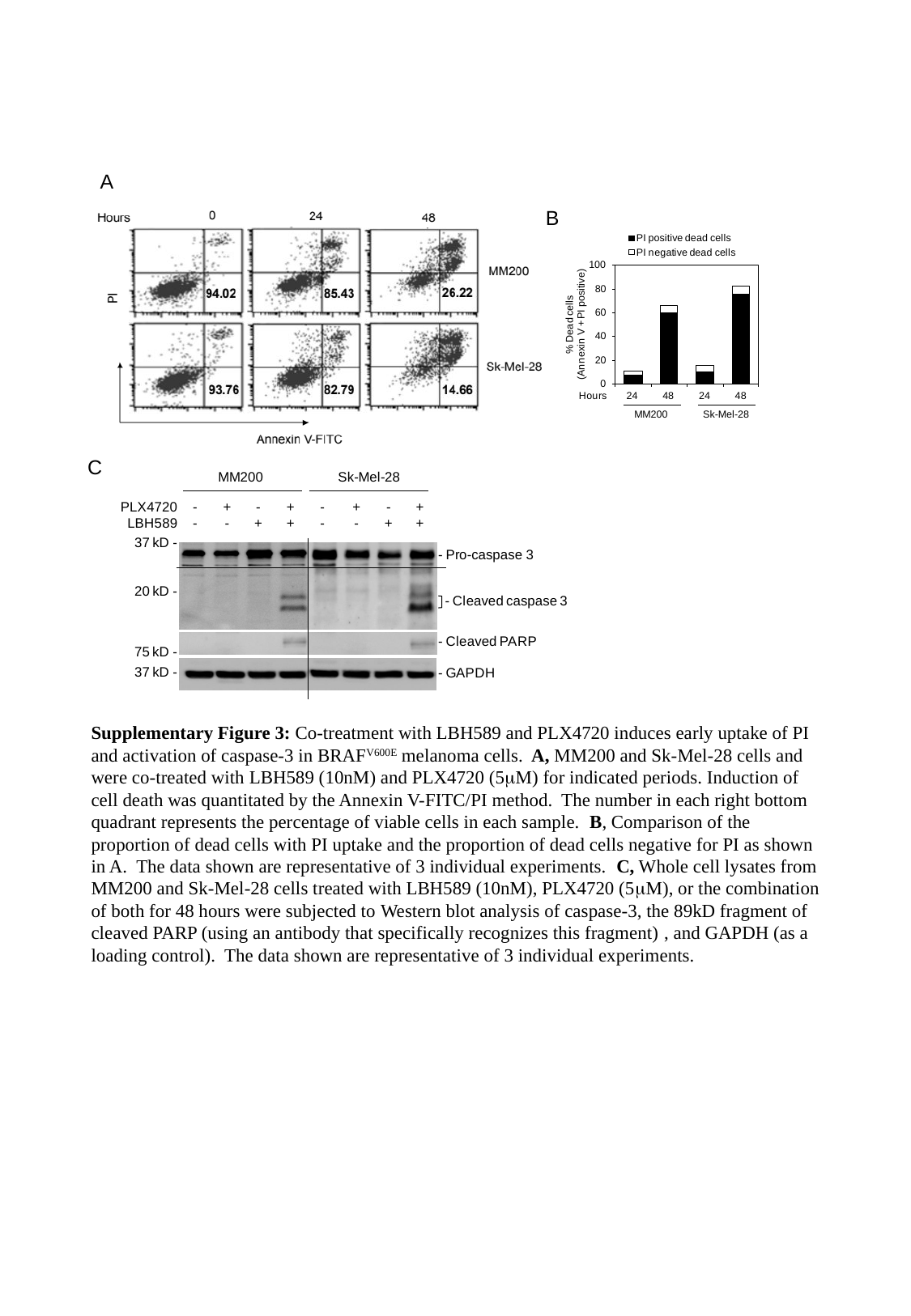

A
B
C
Supplementary Figure 3: Co-treatment with LBH589 and PLX4720 induces early uptake of PI and activation of caspase-3 in BRAFV600E melanoma cells. A, MM200 and Sk-Mel-28 cells and were co-treated with LBH589 (10nM) and PLX4720 (5μM) for indicated periods. Induction of cell death was quantitated by the Annexin V-FITC/PI method. The number in each right bottom quadrant represents the percentage of viable cells in each sample. B, Comparison of the proportion of dead cells with PI uptake and the proportion of dead cells negative for PI as shown in A. The data shown are representative of 3 individual experiments. C, Whole cell lysates from MM200 and Sk-Mel-28 cells treated with LBH589 (10nM), PLX4720 (5μM), or the combination of both for 48 hours were subjected to Western blot analysis of caspase-3, the 89kD fragment of cleaved PARP (using an antibody that specifically recognizes this fragment) , and GAPDH (as a loading control). The data shown are representative of 3 individual experiments.
